# IgG-Fc glycosylation before and after rituximab treatment in immune thrombocytopenia

**DOI:** 10.1038/s41598-020-59651-7

**Published:** 2020-02-20

**Authors:** David E. Schmidt, Noortje de Haan, Myrthe E. Sonneveld, Leendert Porcelijn, C. Ellen van der Schoot, Masja de Haas, Jaap-Jan Zwaginga, Manfred Wuhrer, Gestur Vidarsson

**Affiliations:** 1 Sanquin Research, Department of Experimental Immunohematology, Amsterdam, The Netherlands and Landsteiner Laboratory, Amsterdam UMC, University of Amsterdam, Amsterdam, The Netherlands; 2 Center for Proteomics and Metabolomics, Leiden University Medical Center, Leiden, The Netherlands; 3 Department of Immunohematology Diagnostics, Sanquin Diagnostic Services, Amsterdam, The Netherlands; 4 Sanquin Research, Center for Clinical Transfusion Research, Leiden, The Netherlands, and Jon J van Rood Center for Clinical Transfusion Science, Leiden University Medical Center, Leiden, The Netherlands; 5 Department of Immunohematology and Blood Transfusion, Leiden University Medical Center, Leiden, The Netherlands

**Keywords:** Molecular medicine, Translational research, Autoimmunity, Antibodies

## Abstract

The interactions of antibodies with myeloid Fcγ receptors and the complement system are regulated by an Asn297-linked glycan in the Fc portion of IgG. Alterations of serum IgG-Fc glycosylation have been reported in various autoimmune diseases, and correlate with treatment response and disease activity. We hypothesized that IgG-Fc glycosylation is altered in immune thrombocytopenia (ITP) and associates with response to anti-CD20 monoclonal antibody treatment (rituximab). IgG-Fc glycosylation was analyzed by liquid chromatography-mass spectrometry. We found that IgG-Fc glycosylation was identical between refractory ITP patients (HOVON64 trial; N = 108) and healthy controls (N = 120). Two months after rituximab treatment, we observed a shift in Fc glycosylation, with a mean 1.7% reduction in galactosylation for IgG1 and IgG4 and a mean 1.5% increase for bisection in IgG1, IgG2/3 and IgG4 (adjusted p < 1.7 × 10^−3^ and p < 2 × 10^−4^, respectively). Neither baseline nor longitudinal changes in IgG-Fc glycosylation after rituximab were associated with clinical treatment response. We conclude that IgG-Fc glycosylation in refractory ITP is similar to healthy controls and does not predict treatment responses to rituximab. The observed changes two months after treatment suggest that rituximab may influence total serum IgG-Fc glycosylation. Overall, our study suggests that the pathophysiology of refractory ITP may differ from other autoimmune diseases.

## Introduction

Immune thrombocytopenia (ITP) is an autoimmune bleeding disease characterized by self-reactive cellular and humoral anti-platelet responses that result in platelet clearance. A hallmark of ITP in adults are IgG-anti-platelet autoantibodies^1^. The diagnosis of ITP is established clinically by exclusion of alternative causes of thrombocytopenia^2^, which is thought to result in diagnostic heterogeneity. Rituximab, an anti-CD20 antibody targeting B cells, represents an important second-line treatment, with about 60% of patients responding. The underlying working mechanisms remain incompletely understood^3–5^.

The Fc portion of each heavy chain of an IgG molecule has a single glycosylation site at Asn297. The exact composition of the attached *N*-glycan affects effector functions through modification of the Fc-tail affinity for Fcγ receptors and C1q-mediated complement activation^6^. The total serum IgG-Fc glycosylation is skewed in multiple autoimmune diseases, such as rheumatoid arthritis^7,8^, autoimmune hemolytic anemia^9^, systemic lupus erythematosus^10^, Guillain-Barre syndrome^11^, vasculitis^12,13^ and inflammatory bowel disease^14^. Across these studies, the strongest association with autoimmune diseases is a reduction in Asn-297 galactosylation, followed by sialylation, which are linked because galactosylated glycan structures are substrates for sialyltransferases^6^. Moreover, GWAS-identified loci that regulate Fc *N*-glycosylation are strongly associated with susceptibility to autoimmune diseases^15^.

In rheumatoid arthritis, extensive data indicate that a low total IgG-Fc galactosylation correlates with symptom onset and disease activity^16–20^. The low galactosylation reverts towards normal levels during treatment or spontaneous recovery, as observed during pregnancy^21–23^. In Guillain-Barrè syndrome and Kawasaki disease, IVIg-responses correlate with IgG-Fc glycosylation profiles before treatment as well as with treatment-induced changes of Fc glycosylation^11,24^.

Immunotherapy with immunomodulating biologicals can affect the IgG glycosylation, reflecting direct and indirect effects on the immune system. For instance, the reduced IgG galactosylation in rheumatoid arthritis, psoriatic arthritis and spondyloarthropathy were found to revert towards normal levels after anti-TNFα immunotherapy^23,25,26^, and this correlated with associated changes in CRP levels^27^. The effect of B cell depleting antibody therapy, such as rituximab, has however not been studied.

In the present study, we tested if serum IgG-Fc glycosylation is altered in ITP, similar to other autoimmune diseases. We further hypothesized that baseline or dynamic changes of IgG-Fc glycosylation may predict treatment response to rituximab.

## Results

**Figure 1:**
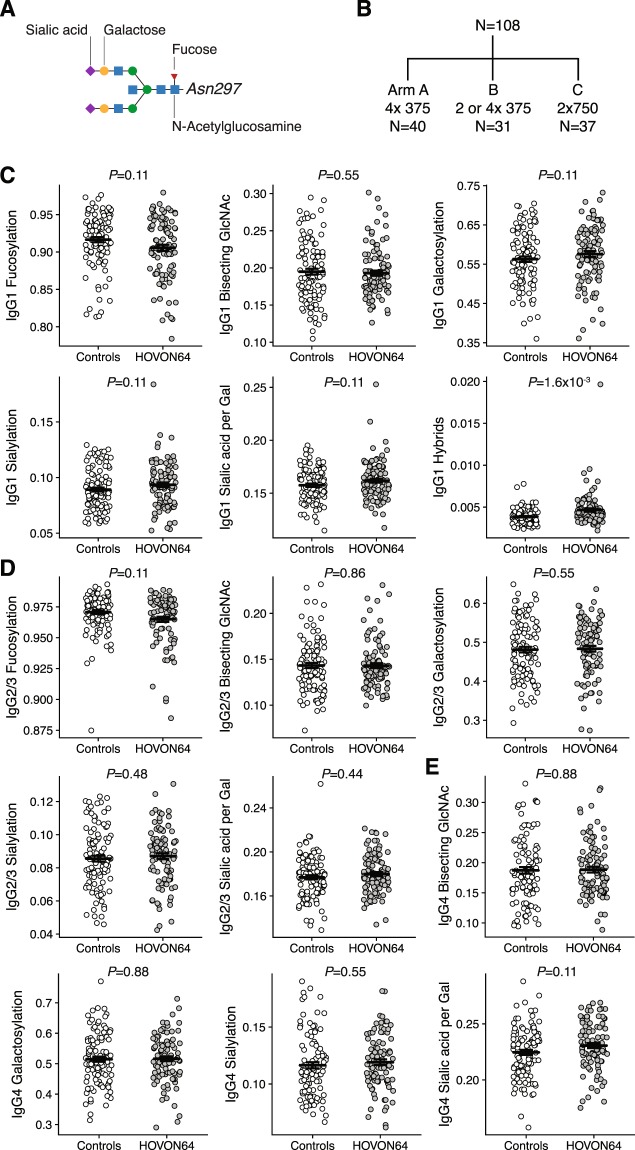
IgG1-Fc glycosylation is similar between patients with immune thrombocytopenia and healthy controls. (**A**) An example of complex-type IgG-linked Fc glycan as found in our samples: a diantennary, digalactosylated, disialylated N-glycan carrying a core fucose and a bisecting N-acetylglucosamine (GlcNAc). (**B**) Patients with refractory ITP (N = 108) were randomized to three treatment schemes of rituximab (detailed in method section). (**C**–**E**) Overview of IgG1-, IgG2/3- and IgG4-Fc glycan traits in ITP (N = 108) and healthy controls (N = 120). Data are individual patient measurements (mean ± SEM). Indicated p-values are given for the effect of ITP in a linear model of the respective Fc glycan, adjusted for covariates age and sex, and corrected for multiple comparisons.

The *N*-linked Fc glycan composition at position Asn297 found in all IgG subclasses (Fig. 1A) was analyzed in 108 ITP patients and 120 age- and sex-matched healthy controls. Patients were randomized to one of three treatment schemes (detailed in methods; Fig. 1B)^28^. IgG2 and IgG3 results are analyzed together (IgG2/3) because of overlapping peptide moieties^29^.

Unlike observations made for other autoimmune diseases, ITP patients showed a total IgG-Fc glycosylation similar to healthy controls (Fig. 1C–E). In particular, there was no reduction of IgG1 galactosylation in ITP. Importantly, we did observe the expected age-dependent differences in healthy controls as well as excellent reproducibility in technical controls (N = 25, coefficient of variation, 4.9%; data not shown). The only statistically significant finding was a minor absolute difference in the level of IgG1 hybrid-type Fc glycans (Fig. 1C), with higher levels in the patient group. As this hybrid glycan-type, containing a mannose on the α1,6 arm of the glycan, comprises only a very small fraction of the Fc glycan repertoire of total serum IgG (generally <1%), it is unlikely that this is of functional significance.

**Figure 2:**
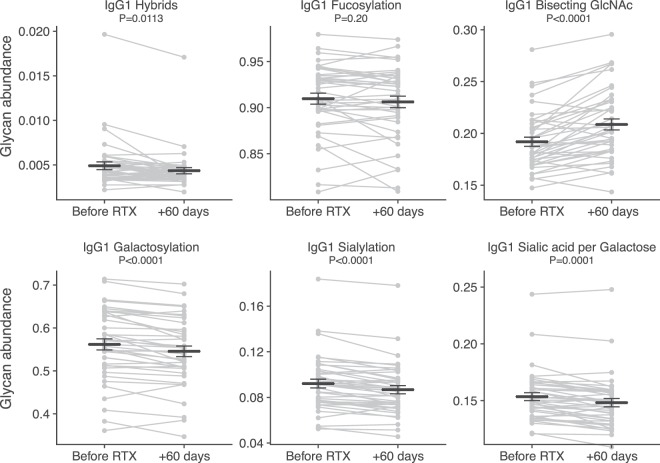
Rituximab treatment has complex effects on IgG1-Fc glycosylation. Paired samples of immune thrombocytopenia patients (N = 41) were assessed before and 60 days after rituximab treatment. Data are individual patient measurements (mean ± SEM). p-values are given for a paired t-test, corrected for multiple comparisons.

To evaluate the impact of rituximab on Fc glycosylation, we compared paired samples obtained before and 60 days after start of treatment (N = 41; Fig. 2), which is well beyond the IgG half-life of 21 days^30^. We observed statistically significant changes in the composition of Asn297-linked Fc glycans of most IgG subclasses, but not for IgG1 fucosylation, IgG2/3 fucosylation or IgG2/3 galactosylation (Fig. 2; IgG2/3 and IgG4 data not shown). There were no differences in the direction of change between the allocated treatment regimens. The observed changes on absolute glycan levels were minor: the largest effect after rituximab treatment was a mean 1.7% reduction in IgG1 and IgG4 galactosylation, followed by a mean 1.5% increase in Fc-bisection for all IgG subclasses (adjusted p < 1.7 × 10^−3^ and p < 2 × 10^−4^, respectively).

**Figure 3:**
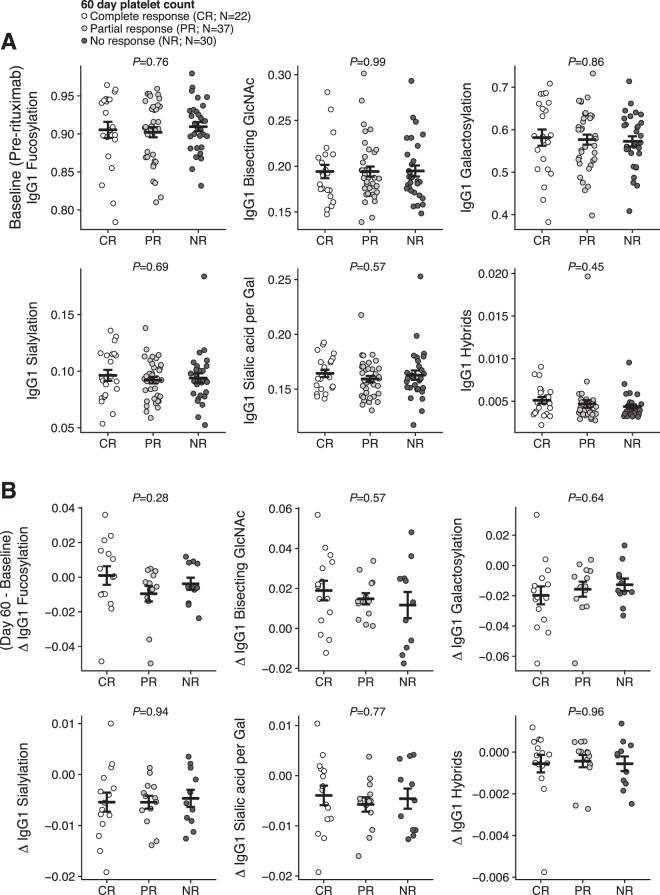
IgG1-Fc glycosylation is not associated with response to rituximab. (**A**) Baseline levels of IgG1-Fc glycans and response to rituximab 60 days after treatment. (**B**) Dynamic changes (day 60 minus baseline levels) in IgG1-Fc glycans are not associated with response to rituximab in paired samples. Data are individual patient measurements (mean ± SEM). p-values are given for ANOVA (adjusted for age and sex for the baseline levels). This was a secondary, exploratory analysis and p-values were not corrected for multiple comparisons.

Baseline IgG glycosylation could be predictive of response to immunomodulatory therapy, as shown for Guillain-Barrè syndrome or Kawasaki disease^11,24^. Therefore, we assessed if IgG-Fc glycosylation were associated with a clinical response to rituximab. Of patients with available platelet response data two months after rituximab, 22 patients had a complete response (CR; platelet count ≥100 × 10^9^ L^−1^), 37 a partial response (PR; platelet count ≥30 × 10^9^ L^−1^), and 30 no response (NR)^31^. We observed no association between levels of IgG1-Fc glycosylation traits before rituximab administration and platelet responses (Fig. 3A).

ITP is a heterogeneous disorder and a subgroup of ITP patients has no anti-platelet antibodies and responds differently to rituximab treatment^5^. However, total IgG-glycosylation levels between antibody positive and negative patients were similar, and there was no association between IgG-glycosylation with response for either of these subgroups (Supplementary Table). Moreover, in patients with paired samples before and 2 months after rituximab treatment (CR, N = 15; NR, N = 11; PR, N = 13), there was no relationship between baseline levels or change in IgG1-Fc glycans and platelet response (Fig. 3B).

## Discussion

In contrast to our hypothesis, we unexpectedly found that refractory ITP patients showed no skewing of IgG-Fc glycosylation, which is observed - in particular with a low total serum IgG-galactosylation - in other autoimmune diseases^9–11,13,16^. Our findings may suggest that the pathophysiological mechanisms in ITP are different from other antibody-mediated autoimmune diseases, where IgG-glycosylation shows clear associations with disease onset and progression(in particular also autoimmune hemolytic anemia^9^).

The exact mechanism by which IgG Fc-galactosylation and sialylation is reduced in autoimmune diseases and inflammation is unknown. Lowered IL-6 has been associated with decreased IgG Fc-galactosylation^32,33^. As IL-6 is a primary cytokine of the acute-phase response and subsequent inflammation, the possibility exists that Fc-galactosylation is indirectly associated with inflammation. Ultimately, the increased serum levels and B cell expression of beta-galactosidase, an enzyme that cleaves terminal galactose residues, might be one of the involved pathways^34^. Moreover, IL-21 and IL-22 produced by Th17 cells may instruct B cells to reduce expression of St6Gal1, which leads to asialylated IgG species^18^. Why these pathways are apparently not affected in ITP is unclear. We speculate that heterogeneity in the clinical background of ITP may be one of the reasons that we could not detect any change in IgG Fc-glycosylation. First, it is known that a proportion of patients initially diagnosed as ITP may have other, non-immune causes of thrombocytopenia^35^. Previous work by our group and others has indicated that treatment-refractory patients may not have any detectable anti-platelet antibodies in the first place^5,36^, and CD8-T cell mediated platelet clearance could be an alternative mechanism that maintains thrombocytopenia^37,38^.

We report for the first time that rituximab treatment alters serum IgG glycosylation. The observed changes were minor, the most pronounced being an increase of bisecting GlcNAcs and a decrease in galactosylation after treatment. These changes in IgG-Fc glycosylation are perhaps not surprising as rituximab depletes circulating and lymph-node resident CD20^+^ B cells for >2–6 months^39,40^. Although the precise regulation of IgG-Fc glycosylation is still incompletely elucidated, it is clear that B cell glycosyltransferases regulate the glycosylation in an antigen-specific manner^41–44^. Importantly, rituximab does not affect IgG levels^40,45,46^, suggesting that these are maintained by CD20-negative plasma cells. Although there were effects of rituximab on IgG glycosylation, these changes did not associate with the clinical response to rituximab (Fig. 3).

Therefore, in ITP, it is likely that rituximab exerts its immunomodulatory effects through other mechanisms, e.g. IgM positive B cells, an influence on T cell balance^47^, the induction of regulatory B cells^48^, or simply through reduction of anti-platelet antibodies in ITP^5^. Whether glycosylation of those pathogenic antibodies is affected is still unknown and remains challenging to investigate due to the minute amount of these antibodies.

Our study focused on refractory ITP patients who all had long-standing and ongoing disease. This implied that they had already received first-line treatment with corticosteroids, which might have affected changes in IgG glycosylation. However, patients treated with high-dose corticosteroids within three weeks were not eligible for trial participation. One study found that corticosteroids reduced IgG1 Fc-galactosylation and -sialylation slightly in patients with inflammatory bowel disease^14^. Accordingly, if corticosteroid would have affected the present results, it would have been expected to result in lowered IgG1 Fc-galactosylation and -sialylation, which we did not observe. A further limitation of our study was that we did not have an untreated control group, and the changes 60 days after rituximab administration may theoretically represent the natural course of disease and not rituximab treatment *per se*.

In conclusion, IgG-Fc glycosylation in ITP patients was similar to healthy controls, and no association was found with treatment outcomes after rituximab. Rituximab treatment may have an effect on total serum IgG Fc-glycosylation, as observed two months after treatment. Our data suggest that the pathophysiology of refractory ITP may differ from other autoimmune diseases.

## Methods

### Study subjects

Adult refractory ITP samples were included from the Dutch Hemato-Oncology Cooperative Group (HOVON) HOVON64 study^28^, a randomized controlled multicenter trial in the Netherlands. In brief, patients with relapsed or refractory ITP above 18 years who completed first-line treatment with corticosteroids and had at least 2 platelets counts ≤30 × 10^9^ L^−1^ and a WHO performance status of 0–2 were eligible for inclusion. Patients were not eligible for study entry if they received treatment with pulsed or high dose corticosteroids, IVIG or splenectomy less than 3 weeks before randomization. Further exclusion criteria included prior treatment with rituximab, active grade 3 or 4 bleeding (by NCI CTCAE v3.0 criteria), the presence of malignancies, systemic infections or systemic autoimmune disorders. Patients were randomized into three arms to receive (A) 4 weekly doses of 375 mg/m^2^ body surface area rituximab, (B) 2 weekly doses of 375 mg/m^2^ rituximab, with potential extension to receive 2 additional doses in case of no response, or (C) 2 weekly doses of 750 mg/m^2^ rituximab. The study was approved by the medical ethical committee of Academic Medical Center Amsterdam and all participants gave written informed consent. Splenectomized patients were excluded for the present study. The median age of the patients was 52 years (interquartile range [IQR], 35–64) and 58% were female. The median time between ITP diagnosis and start of rituximab was 1.06 years (IQR 0.34–3.14). Control samples were obtained from age- and sex-matched healthy Dutch blood donors by antecubital venipuncture before a blood donation (Sanquin, Amsterdam; The Netherlands). The study was performed under Dutch national guidelines (Human Tissue and Medical Research: Code of conduct for responsible use; https://www.federa.org/codes-conduct) and conducted in accordance with the Declaration of Helsinki. All analyses were performed on coded, de-identified data. Stored serum or plasma samples (−20 °C) were used for analyses of IgG-Fc glycosylation by LC-MS.

### IgG-Fc glycosylation

IgG was isolated using protein G affinity beads (GE Healthcare, Uppsala, Sweden), as previously described^9^. After washing the beads, IgG was eluted with formic acid, dried in a vacuum concentrator, trypsin digested and stored at −20 °C. Analysis of the tryptic IgG-Fc glycopeptides was performed with nanoLC-reversed phase-electrospray-quadrupole time-of-flight (QTOF)-mass spectrophotometry (MS) on an Ultimate 3000 RSLCnano system (Dionex Corporation, Sunnyvale, CA) coupled to an Impact QTOF-MS (Bruker Daltonics, Bremen, Germany)^49,50^. The nanoLC-MS data was processed using LaCyTools^51^, as previously described^9^.

### Statistical analysis

Statistical analyses were performed in R version 3.4.1. A sample size calculation was performed assuming similar differences in IgG1 galactosylation as observed before in autoimmune hemolytic anemia^9^, with a mean difference of 10% and standard deviation of 8%. For a power of 0.9 and a significance level of 0.001, 30 individuals were required in each group. Additional samples were included based on availability. Due to the age and sex dependency of IgG glycans^52^, groups were compared by constructing a linear model for each glycan, adjusted for age and sex as covariate. Paired samples were evaluated by a paired t-test. Reported p-values for the primary analysis (Figs. 1 and 2) were adjusted for multiple testing by false discovery rate (FDR). P-values in the secondary analysis (Fig. 3), using ANOVA, were not adjusted. A two-tailed p-value < 0.05 was considered statistically significant.

## Supplementary information

Supplementary Information.
