# Supplementary material for: IgG-Fc glycosylation before and after rituximab treatment in immune thrombocytopenia
Source: Sci Rep. 2020 Feb 20;10:3051. doi: 10.1038/s41598-020-59651-7 (PMC7033207; doi:10.1038/s41598-020-59651-7)
Supplement: Supplementary file 1 — Supplementary Information. [file 41598_2020_59651_MOESM1_ESM.pdf]

Online Supplement

IgG-Fc glycosylation before and after rituximab treatment in immune thrombocytopenia

David E. Schmidt, Noortje de Haan, Myrthe E. Sonneveld, Leendert Porcelijn,

C. Ellen van der Schoot, Masja de Haas, Jaap-Jan Zwaginga,

Manfred Wuhrer, Gestur Vidarsson

Supplementary Table. Subgroup analyses.

Baseline levels	Antibody negative (N=28)	Relative change Antibody positive (N=46)
IgG1 Fucosylation	0.95 ± 0.01	-0.01 ± 0.01
IgG1 Bisection	0.15 ± 0.01	0.00 ± 0.00
IgG1 Galactosylation	0.73 ± 0.02	-0.01 ± 0.01
IgG1 Sialylation	0.13 ± 0.01	0.00 ± 0.00
IgG1 Galactosylation per sialic acid	0.18 ± 0.01	0.00 ± 0.01

Mean±SE. No antibody data available at baseline for 29 patients.

	Day 60 response		
**Baseline levels**	**CR (N=25)**	**Relative change PR (N=38)**	**Relative change NR (N=31)**
IgG1 Fucosylation	0.93 ± 0.01	0.00 ± 0.01	0.00 ± 0.01
IgG1 Bisection	0.15 ± 0.01	0.00 ± 0.01	0.00 ± 0.01
IgG1 Galactosylation	0.72 ± 0.02	-0.01 ± 0.02	0.00 ± 0.01
IgG1 Sialylation	0.12 ± 0.01	-0.01 ± 0.01	0.00 ± 0.01
IgG1 Galactosylation per sialic acid	0.18 ± 0.01	-0.01 ± 0.01	0.00 ± 0.01

Mean±SE. No response data for 9 patients.

	Antibody positive patients, Day 60 response		
**Baseline levels**	**CR (N=18)**	**Relative change PR (N=14)**	**Relative change NR (N=9)**
IgG1 Fucosylation	0.92 ± 0.02	-0.01 ± 0.02	0.01 ± 0.02
IgG1 Bisection	0.15 ± 0.02	0.01 ± 0.01	0.00 ± 0.01
IgG1 Galactosylation	0.74 ± 0.03	-0.01 ± 0.02	0.00 ± 0.03
IgG1 Sialylation	0.13 ± 0.01	0.00 ± 0.01	0.00 ± 0.00
IgG1 Galactosylation per sialic acid	0.18 ± 0.01	0.00 ± 0.01	0.00 ± 0.00

Mean±SE. No response data for 5 patients.

	Antibody negative patients, Day 60 response		
**Baseline levels**	**NR (N=13)**	**Relative change PR (N=10)**	**Relative change CR (N=3)**
IgG1 Fucosylation	0.96 ± 0.03	-0.01 ± 0.02	0.00 ± 0.03
IgG1 Bisection	0.15 ± 0.02	0.00 ± 0.01	0.01 ± 0.02
IgG1 Galactosylation	0.70 ± 0.04	0.01 ± 0.03	-0.01 ± 0.04
IgG1 Sialylation	0.12 ± 0.02	0.00 ± 0.01	0.00 ± 0.02
IgG1 Galactosylation per sialic acid	0.17 ± 0.02	0.00 ± 0.01	0.00 ± 0.02

Mean±SE. No response data for 2 patients.
